# Hydrogen bonding in cocrystals of *trans*-Ni^II^(hfac)_2_(H_2_O)_2_

**DOI:** 10.1107/S2053229626009460

**Published:** 2026-09-18

**Authors:** Grace L. Clemmer, Caden W. York, Mackenzie G. Gray, Elisabeth M. Fatila, Alex Lovstedt

**Affiliations:** a Department of Chemistry, Indiana University Bloomington, 800 E. Kirkwood Ave., Bloomington, Indiana 47405-7102, USA; b Department of Chemistry and Physics Louisiana Tech University, 1 Adams Blvd Ruston Louisiana 71272-0001 USA; Oak Ridge National Laboratory, USA

**Keywords:** crystal structure, cocrystal, hy­dro­gen-bond network, solvate, chemical vapor deposition precursor

## Abstract

Chemical vapor deposition (CVD) precursors often employ fluorinated β-diketonate ligands bonded to metal atoms; however, these are prone to the formation of hydrates under ambient conditions and form hy­dro­gen-bond net­works which hinder the CVD process. This work investigated the use of 1,2-di­meth­oxy­ben­zene (DMB) as a ligand to prevent the formation of hy­dro­gen-bonding networks. The single-crystal structures of two com­plexes of di­aqua­bis­(1,1,1,5,5,5-hexa­fluoro-4-oxopent-2-en-2-olato-κ^2^*O*,*O*′)nickel(II), *trans*-Ni^II^(hfac)_2_(H_2_O)_2_, were determined, one as a 4/3 hydrate, [Ni(C_5_HF_6_O_2_)_2_(H_2_O)_2_]_3_·4H_2_O, and one as a 1,2-di­meth­oxy­ben­zene (DMB) pentasolvate, [Ni(C_5_HF_6_O_2_)_2_(H_2_O)_2_]·5C_8_H_10_O_2_. The hy­dro­gen-bond networks of these two materials were com­pared to previously reported hy­dro­gen-bond networks seen in Ni^II^(hfac)_2_(H_2_O)_2_. DMB was found to disrupt the formation of an extended hy­dro­gen-bond network without coordinating to the Ni center.

## Abstract 2

Two cocrystals of *trans*-Ni^II^(hfac)_2_(H_2_O)_2_ con­taining cocrystallized water or 1,2-di­meth­oxy­ben­zene were characterized by single-crystal X-ray diffraction. The hy­dro­gen-bonding networks in each cocrystal were com­pared to previously reported structures, revealing that Ni^II^(hfac)_2_(H_2_O)_2_ is capable of forming a variety of hy­dro­gen-bond network motifs depending on the nature of the coformers in the crystal structure. Cocrystallization of 1,2-di­meth­oxy­ben­zene was found to inhibit the formation of extended hy­dro­gen-bond networks.

## Introduction

The drive for improved microelectronic circuits requires the development of methods to fabricate materials with finely tuned films of metal com­pounds. Chemical vapor deposition (CVD) has been used for decades in the production of microelectronics. For CVD precursors, ensuring thermal stability while allowing for low-tem­per­a­ture volatilization are ideal. A common strategy has been to employ fluorinated β-diketonate ligands, such as 1,1,1,5,5,5-hexa­fluoro­acetyl­ace­ton­ate (hfac) (Mishra & Daniele, 2015 ▸). Under ambient conditions, these transition-metal and lanthanide com­plexes form hydrate adducts to the metal center (Binnemans, 2005 ▸; Malandrino *et al.*, 1996 ▸; Malandrino *et al.*, 2006 ▸; Zhang *et al.*, 2008 ▸). These hydrates hinder the sublimation process, pre­sum­ably through the formation of strong inter­molecular hy­dro­gen-bonding networks. Neutral bidentate ligands such as di­meth­oxy­ethane (DME) are capable of displacing coordinated water mol­ecules (Malandrino *et al.*, 1996 ▸; Fatila *et al.*, 2012 ▸) and are capable of forming volatile mononuclear com­plexes, making them amenable to acting as CVD pre­cursors. We sought to determine whether the 1,2-di­meth­oxy­ben­zene (DMB) ligand could successfully displace the aqua ligands in *trans*-Ni(hfac)_2_(H_2_O)_2_. While the Ni(hfac)_2_(DME) and Mn(hfac)_2_(DME) com­plexes have been prepared (Ibrahim *et al.*, 2025 ▸; Gray *et al.*, 2026 ▸), to the best of our knowledge, analogous com­pounds con­taining the DMB ligand have not been isolated. Based on its reduced flexibility and the reduced donating ability of the meth­oxy group, we did not expect it to coordinate as strongly as DME. We were inter­ested in the possibility that DMB could disrupt the hy­dro­gen-bond network around the Ni(hfac)_2_(H_2_O)_2_ com­plex and what structural changes the DMB ligand could facilitate.

## Experimental

### Chemicals and materials

NaHCO_3_ (99.9%) and NiCl_2_·6H_2_O (99.5%) were purchased from Mallinckrodt Chemical Works. 1,1,1,5,5,5-Hexa­fluoro­pentane-2,4-dione (H-hfac) (98%) and *n*-heptane were ob­tain­ed from Thermo Scientific Chemicals. 1,2-Di­meth­oxy­ben­zene (DMB) (veratrole) was purchased from Acros Organics (99%). Ultrapure water (18 MΩ) was used as solvent.

### Synthetic procedure

**Figure 1:**
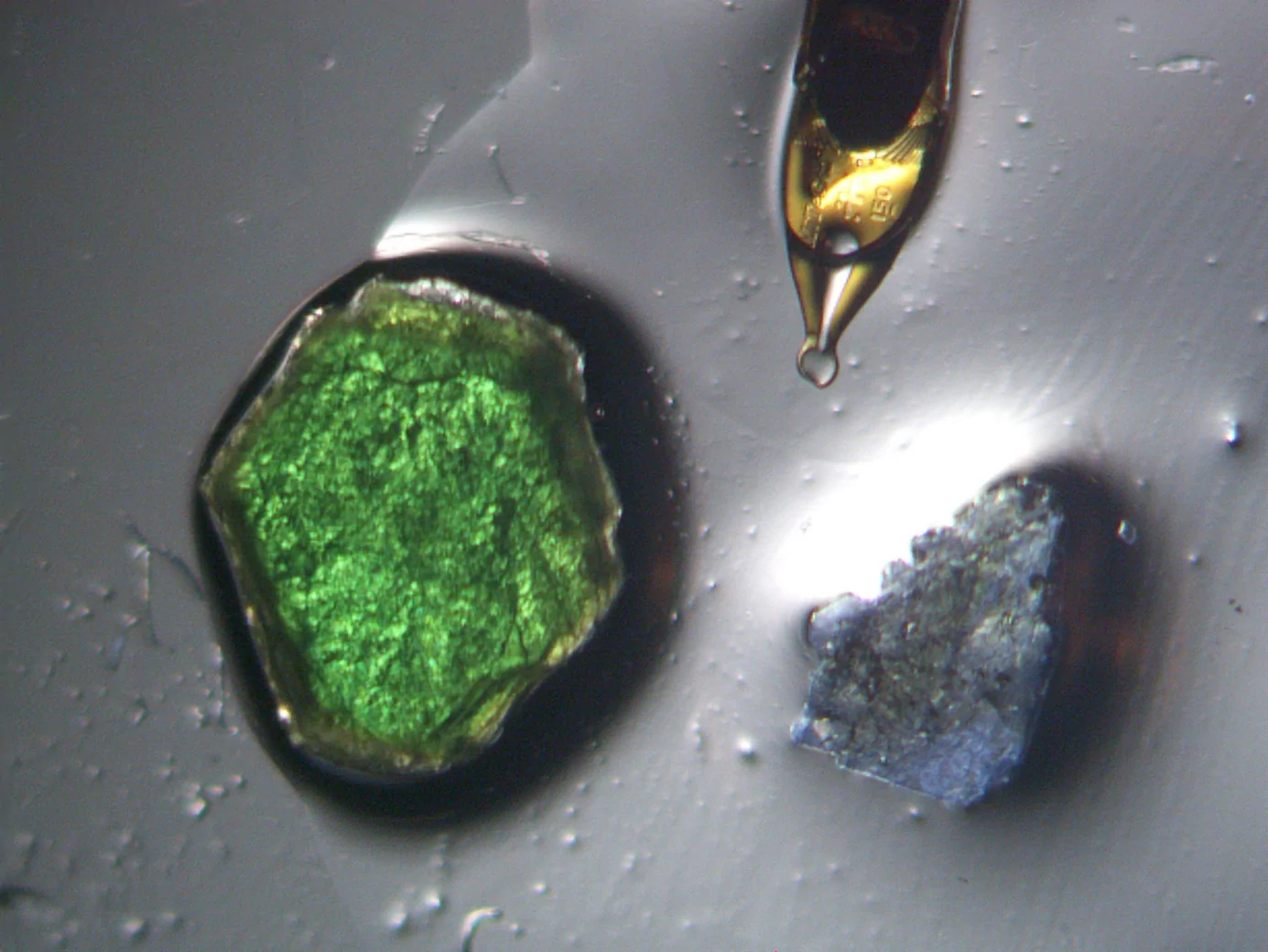
Crystals of **1** (green, left) and **2** (colorless, right). A 150 µm MiTeGen loop is shown for scale. The specimens shown in this image are not the crystals used for the data collection.

NaHCO_3_ (0.5934 g, 7.063 mmol) was dissolved in water (50 ml). An equimolar amount of H-hfac (1 ml, 7 mmol) was then added. The pH was measured to be approximately 6. To this solution, 0.1 g of NaHCO_3_ was added to raise the pH to approximately 7. NiCl_2_·6H_2_O (0.8397 g, 3.533 mmol) was added to the solution. A precipitate immediately formed. The precipitate was filtered off under vacuum. The crude yield was 0.6052 g [33%, 1.189 mmol, assuming a formula of Ni^II^(hfac)_2_(H_2_O)_2_]. The green solid was immediately suspended in *n*-heptane (10 ml) and reacted with 1,2-di­meth­oxy­ben­zene (0.9 ml, 7 mmol). Re­crys­tal­lization *via* slow evaporation yielded colorless and green crystals in the same pot (the yield was not ob­tained). Throughout this article, the green crystals are referred to as **1** and the colorless crystals as **2** (see Scheme 1 and Fig. 1 ▸).

### Crystallographic data collection

Crystal data, data collection and structure refinement details are summarized in Table 1 ▸. H atoms of water mol­ecules were located in the difference map and refined freely with relative isotropic displacement parameters, with the exception of H3*OA*_2 and H3*OB*_2. These atoms were placed *via* the difference map but their positions were not allowed to refine, as otherwise H3*OB*_2 would refine to an unrealistic position. All other H atoms were placed in com­puted locations and refined as riding atoms with relative isotropic displacement parameters. Hirshfeld surface com­positions were calculated using the *CrystalExplorer* software package (Spackman *et al.*, 2021 ▸). The crystal of *trans*-Ni^II^(hfac)_2_(H_2_O)_2_ and water, crystal **1**, for which the data are reported, was found to be a 2-com­ponent non-merohedral twin with an approximate domain ratio of 89:11. The minor domain was rotated from the first domain by 179.9° about an axis in reciprocal space given by the following vector [0.484 0.000 1.000] and an axis in direct space given by the following vector [0.996 0.001 1.000]. The twin law relating the two domains is (in line format): [−0.350 0.004 0.653] [0.000 −1.000 0.000] [1.345 −0.002 0.350].
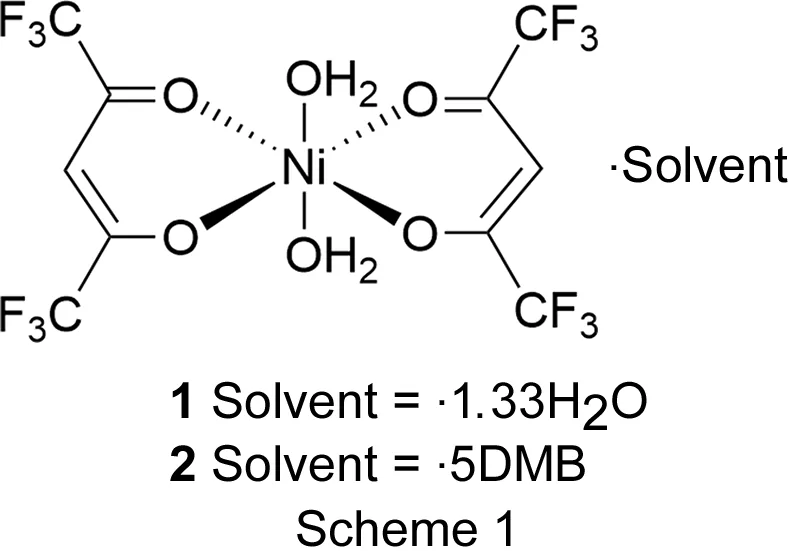


## Results and discussion

### Crystal structure analysis

**Figure 2:**
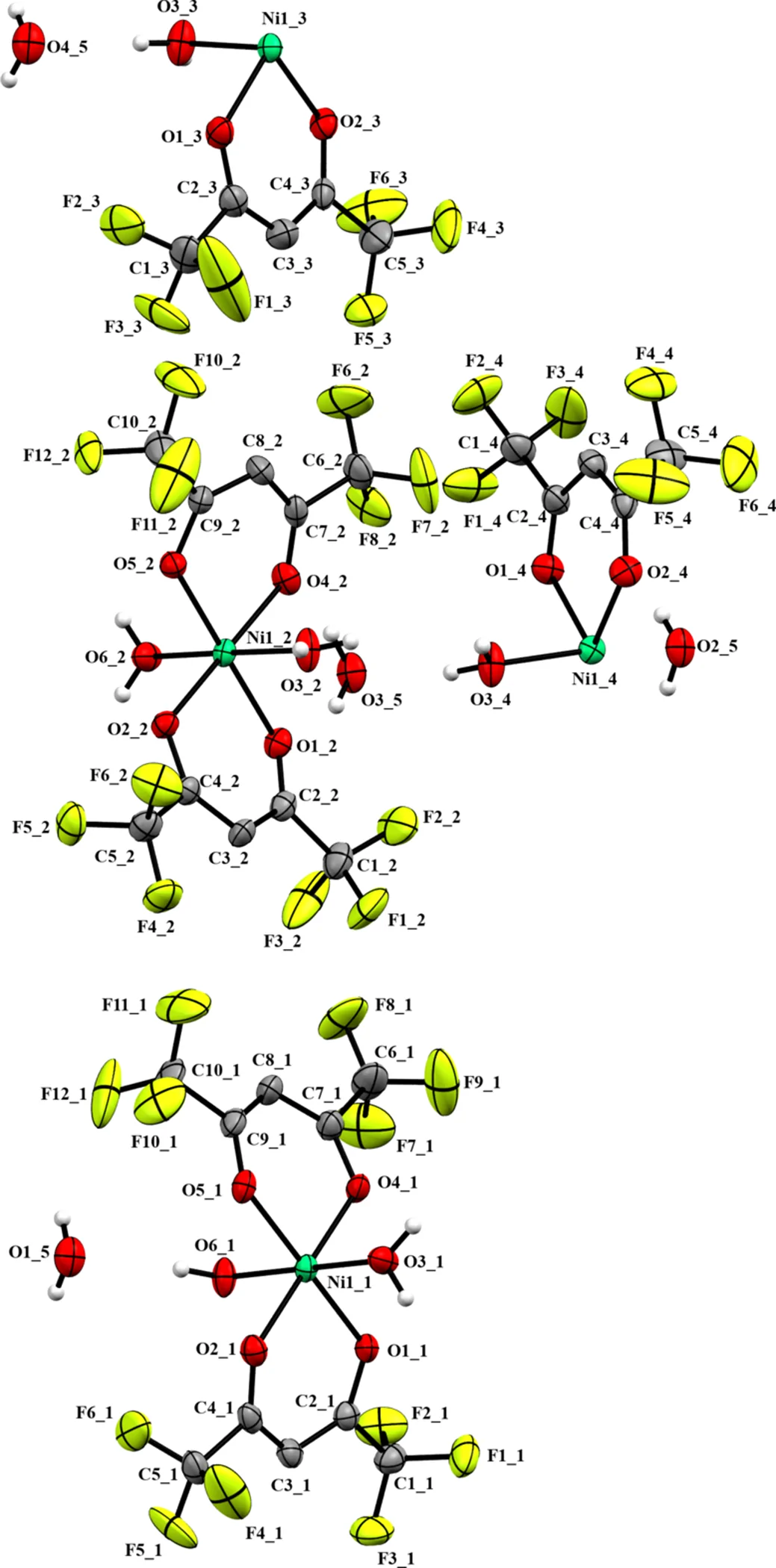
The asymmetric unit of **1**. Only the majorly occupied parts of disordered CF_3_ groups are shown. H atoms not bonded to water have been hidden for clarity. Unless otherwise stated, the following applies to all images of crystal structures in this article: non-H atoms are drawn as displacement ellipsoids at the 50% probability level and H atoms are drawn as fixed size spheres.

**Figure 3:**
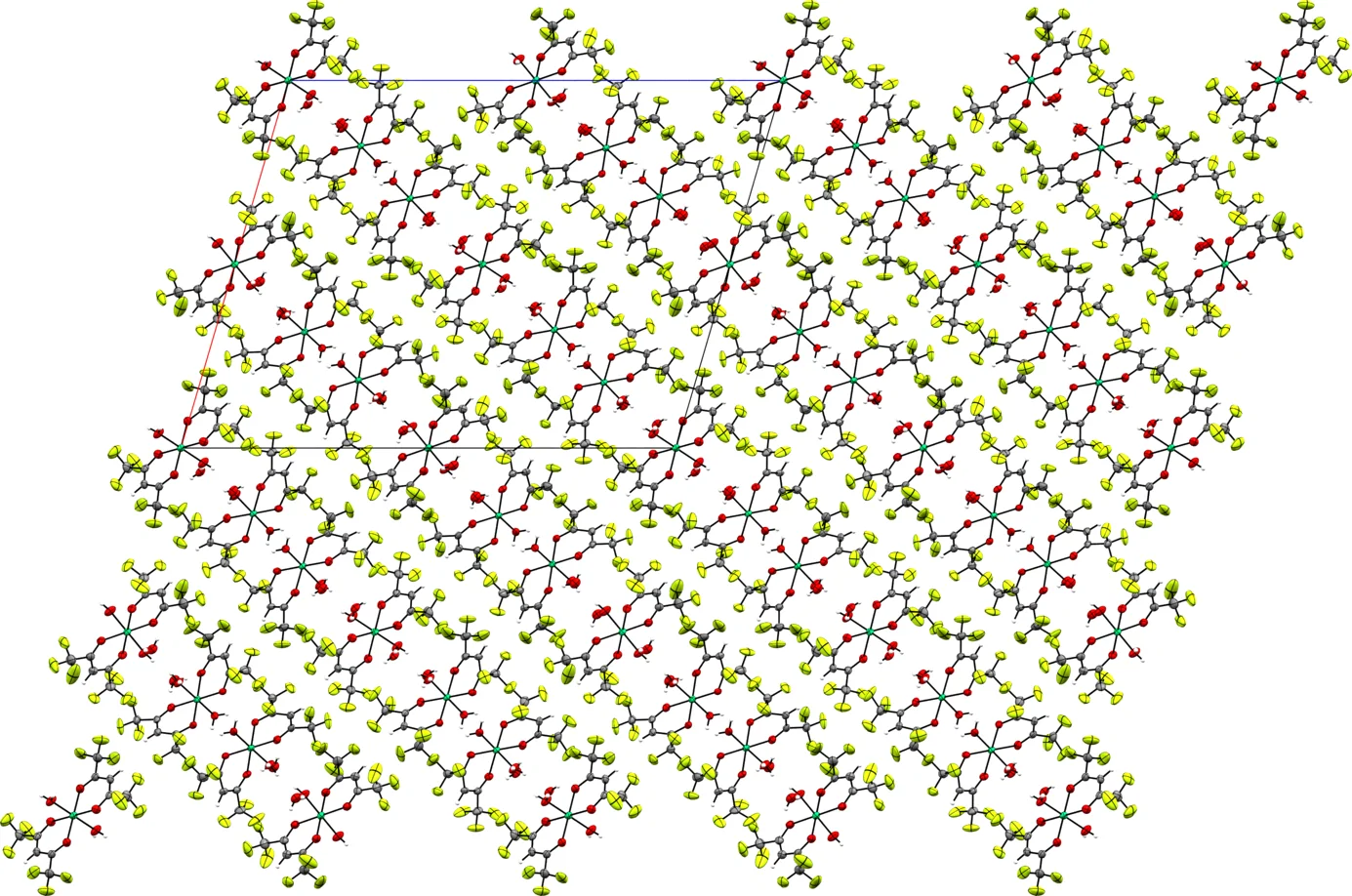
A 2 × 2 × 2 supercell of **1**, viewed along the crystallographic *b* axis, highlighting the alternating layers of hy­dro­gen-bond networks and CF_3_⋯CF_3_ contacts. The red and blue axes of the displayed unit cell are the *a* and *c* axes, respectively. Only the majorly occupied parts of disordered CF_3_ groups are shown.

**Figure 4:**
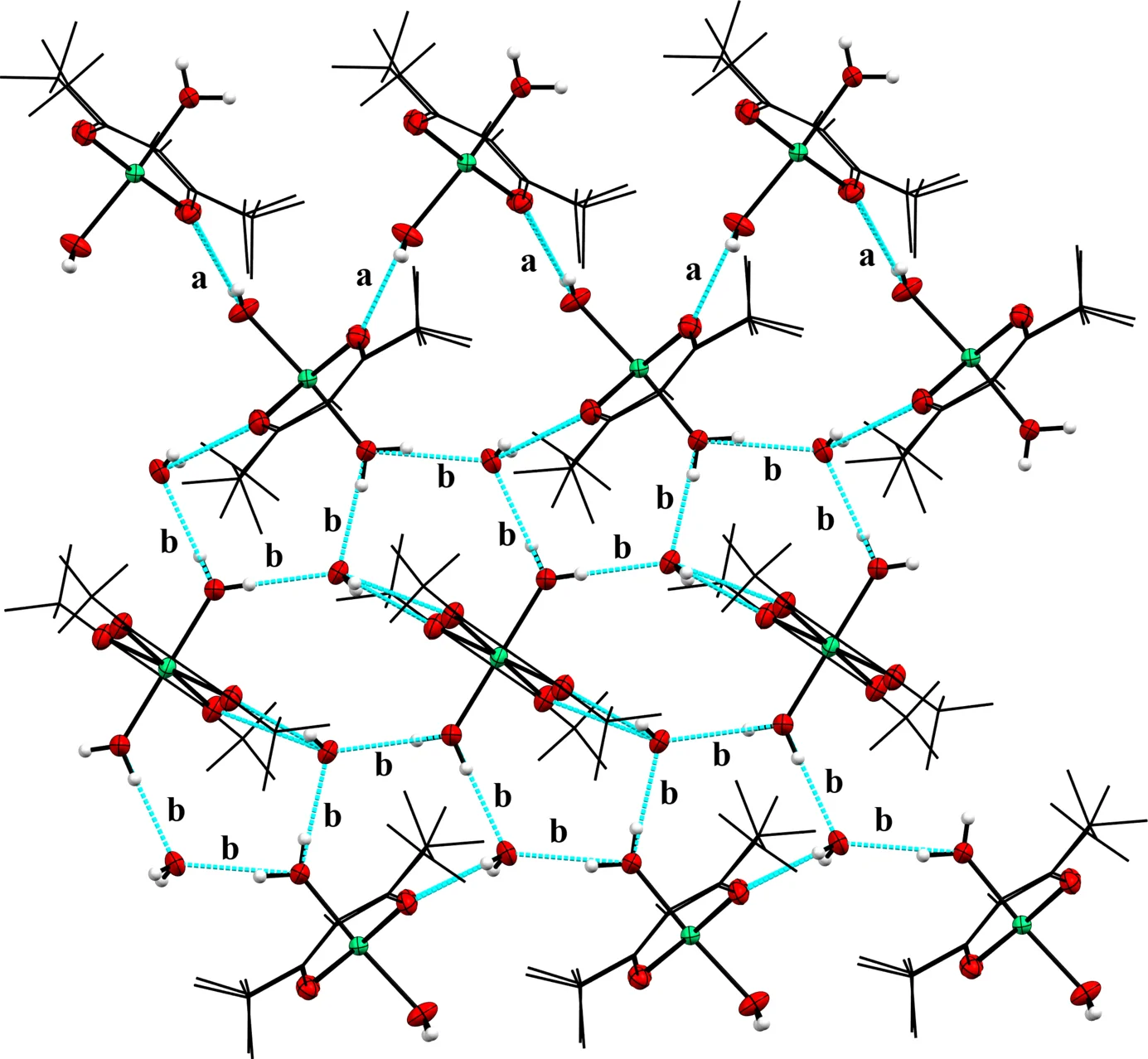
A layer of the hy­dro­gen-bonding network present in **1**, viewed in the [101] direction. In this figure and subsequent figures, hy­dro­gen bonds are repre­sent­ed by dashed blue lines. Hydrogen bonds labeled ‘**a**’ involve an aqua ligand donor and hfac acceptors, while hy­dro­gen bonds labeled ‘**b**’ involve aqua ligand donors and water acceptors. Portions of the hfac ligands are drawn in wireframe for clarity.

The crystal structure of **1** is a cocrystal of *trans*-Ni^II^(hfac)_2_(H_2_O)_2_ and water, and it crystallized in the space group *P*2_1_/*n*. The asymmetric unit con­tains two mol­ecules of the com­plex, as well as two half-mol­ecules in which the Ni atom is located on a crystallographic inversion center (Fig. 2 ▸). There are four cocrystallized water mol­ecules, resulting in a *trans*-Ni^II^(hfac)_2_(H_2_O)_2_ to water stoichiometry of 3:4. Eight of the twelve tri­fluoro­methyl (CF_3_) groups in the asymmetric unit are disordered and modeled in two partially occupied parts. Geometric and rigid bond restraints were applied to both parts of all of the disordered CF_3_ groups to ensure reasonable geometries and anisotropic displacement parameters (ADPs). When necessary, ADP constraints were applied to the F atoms to prevent the refinement of prolate, oblate, or non-positive definite ADPs. Bond distance restraints were applied to the O—H and H⋯H distances for all of the aqua ligands to ensure similar bond lengths and angles between all of the ligands. Similar restraints were applied to the cocrystallized water molecules in the structure. The packing of crystal **1** consists of alternating layers parallel to the [101] direction featuring hy­dro­gen-bonding inter­actions between the aqua ligands and free water mol­ecules, and sheets of CF_3_⋯CF_3_ inter­actions (Fig. 3 ▸ and Table 2 ▸). There are two primary hy­dro­gen-bonding motifs that make up the hy­dro­gen-bonding layers, which will be categorized using graph-set notation (Etter *et al.*, 1990 ▸). In the first type (Fig. 4 ▸, **a**), one of the aqua ligands on the *trans*-Ni^II^(hfac)_2_(H_2_O)_2_ com­plex forms an 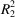
(6) hy­dro­gen-bonded ring with the two O atoms of a hfac ligand on an adjacent Ni^II^(hfac)_2_(H_2_O)_2_ com­plex. The aqua ligand at the other end of the com­plex donates a hy­dro­gen bond to two of the cocrystallized water mol­ecules. In the second type of hy­dro­gen-bond motif, both of the aqua ligands on a *trans*-Ni^II^(hfac)_2_(H_2_O)_2_ unit act as hy­dro­gen-bond donors to cocrystallized water mol­ecules, forming 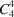
(6) hy­dro­gen-bond chains (Fig. 4 ▸, **b**). The cocrystallized water mol­ecules in this layer also form 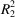
(6) hy­dro­gen-bonded rings with the O atoms of adjacent hfac ligands. This leads to a general packing scheme in which one layer of an aqua–hfac 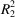
(6) hy­dro­gen-bond network is followed by two layers of 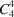
(6) aqua–water hy­dro­gen-bond networks. All cocrystallized water mol­ecules act as hy­dro­gen-bond donors to the O atoms of hfac ligands and as acceptors from aqua ligands. This com­plex hy­dro­gen-bonding network explains the large num­ber of mol­ecules in the asymmetric unit of the crystal. As shown in Fig. 1 ▸, crystals of **1** were large and it was necessary to cut the crystals for data collection. Obtaining a high-quality single crystal of a suitable size proved to be difficult, as the crystals tended to slip rather than split cleanly when being cut. This resulted in streaks in the diffraction pattern, and also made it difficult to select a quality single crystal. Data collection was attempted on several crystals before a satisfactory structure solution could be ob­tained. The crystal packing offers a possible explanation for the macroscopic slipping behavior of the crystals. Based on the weak inter­molecular forces of CF_3_⋯CF_3_ contacts, it can be speculated that when the crystals were being cut for mounting the slipping occurred along the areas of CF_3_⋯CF_3_ inter­actions, although this was not confirmed by experiment.

**Figure 5:**
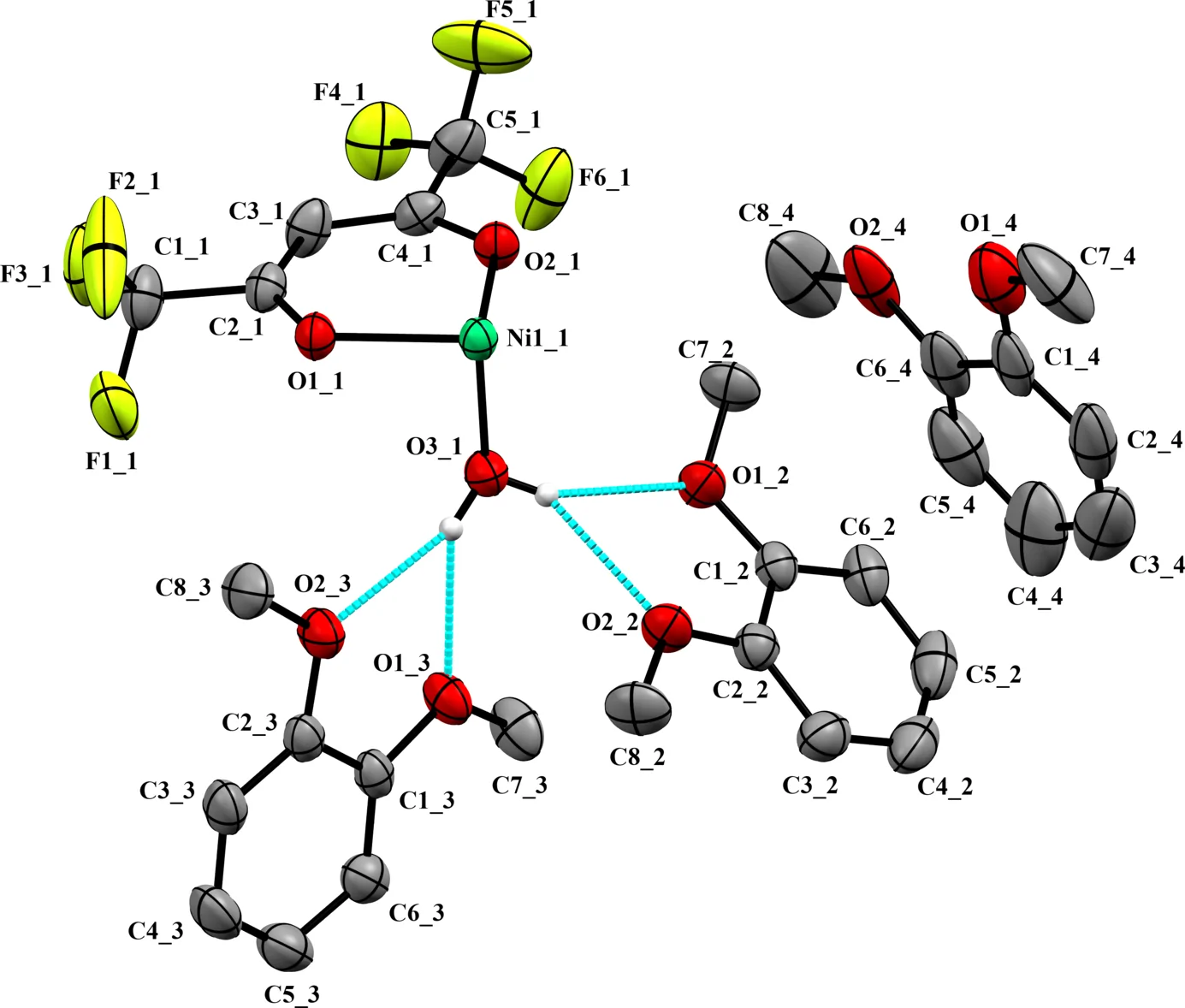
The asymmetric unit of **2**. The DMB mol­ecule in Residue 4 is disordered. Only the majorly occupied disordered parts are shown. H atoms not bonded to water have been hidden for clarity.

**Figure 6:**
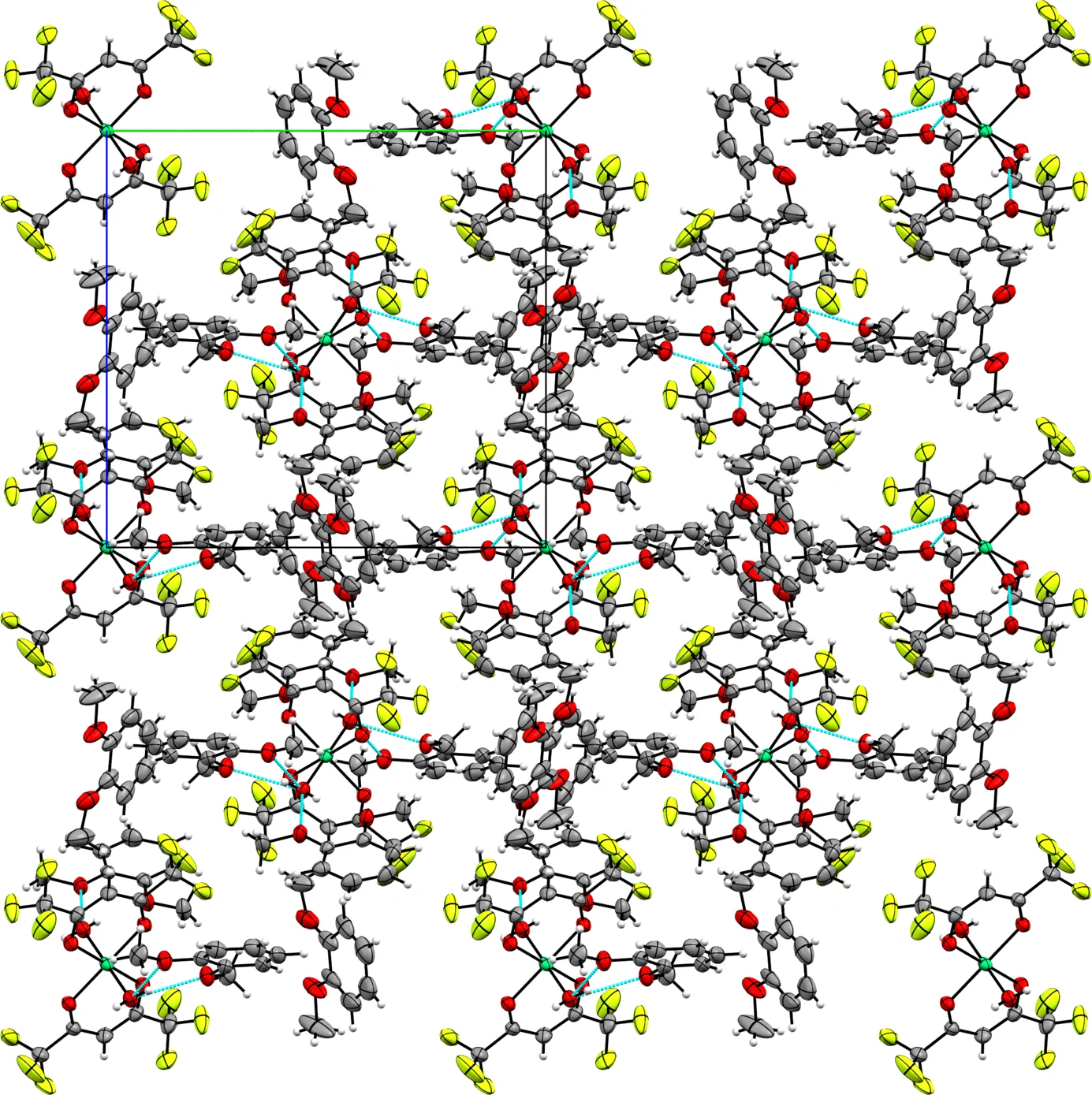
A 2 × 2 × 2 supercell of **2**, viewed along the crystallographic *a* axis. The *b* and *c* axes are shown in green and blue, respectively. Minorly occupied portions of disordered parts have been hidden for clarity.

Crystal **2** is a cocrystal of *trans*-Ni^II^(hfac)_2_(H_2_O)_2_ and DMB, and crystallized in the space group *P*2_1_/*c*. The Ni atom is situated on a crystallographic inversion center, leading to half of the mol­ecule being crystallographically unique. Within the asymmetric unit, the aqua ligand acts as a hy­dro­gen-bond donor to two cocrystallized DMB mol­ecules, with both of the O atoms on the DMB acting as acceptors to one of the H atoms of the aqua ligand in an 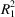
(5) manner (Fig. 5 ▸ and Table 3 ▸). Each of the CF_3_ groups of the complex are disordered and were modeled in two partially occupied parts using geometric and rigid bond restraints to ensure realistic geometries and ADPs. An ADP constraint was used to prevent atom F3′_1 from having prolate ADPs. There is an additional cocrystallized DMB mol­ecule that is located in a cavity on a crystallographic inversion center and is only half occupied within the asymmetric unit, giving a *trans*-Ni^II^(hfac)_2_(H_2_O)_2_ to DMB stoichiometry of 1:5. The DMB mol­ecule located on the inversion center is disordered and was modeled in two partially occupied general positions, with occupancies that add up to 50%. The two disordered parts were modeled using a geometric restraints based on the ordered DMB molecule in Residue 3. A rigid bond restraint was applied to both parts of the disordered DMB molecule. In the unit cell, the Ni atoms of the *trans*-Ni^II^(hfac)_2_(H_2_O)_2_ com­plexes sit on the inversion centers located at 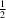, 0, 0 and 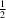, 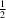, 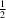, with all of the Ni atoms in a plane. The disordered free DMB mol­ecule sits on the inversion centers between the metal com­plexes at 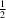, 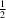, 0 and 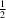, 0, 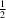 (Fig. 6 ▸). In the packing of the crystal, the *trans*-Ni^II^(hfac)_2_(H_2_O)_2_ com­plexes and associated hy­dro­gen-bond­ed DMB mol­ecules form contacts with the free DMB mol­ecule in the crystallographic *b* and *c* directions, and contact adjacent com­plexes in the *a*, [011], and [0
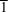
1] directions (Fig. 6 ▸).

Like with **1**, crystals of **2** were large and needed to be cut to obtain an appropriate size for data collection. Contrary to **1**, the crystals of **2** cleaved cleanly, likely because the crystal structure lacks the layers of CF_3_⋯CF_3_ inter­actions present in com­pound **1**. In fact, there are no F⋯F short contacts (defined as a distance less than the sum of the van der Waals radii of the two atoms) in the crystal packing of **2**, and all of the close contacts to the *trans*-Ni^II^(hfac)_2_(H_2_O)_2_ com­plex are with DMB mol­ecules. The chemical environments of the metal com­plexes in the two crystal structures were investigated using Hirshfeld surface analysis, a useful method for enumerating the differences in packing environments between different mol­ecules in crystal structures (Spackman & Jayatilaka, 2009 ▸). Hirshfeld surfaces were com­puted for the *trans*-Ni^II^(hfac)_2_(H_2_O)_2_ com­plexes in **1** and **2**. Table 4 ▸ gives the surface com­positions for the metal com­plexes in each of the structures. Each of the four *trans*-Ni^II^(hfac)_2_(H_2_O)_2_ mol­ecules in the structure of **1** have very similar surface com­positions, with F⋯F contacts being the largest contributor to the Hirshfeld surface. In contrast, the *trans*-Ni^II^(hfac)_2_(H_2_O)_2_ com­plex in **2** has almost no F⋯F contacts, making up less than 1% of the Hirshfeld surface. The com­plex in **2** has about double the F⋯H/H⋯F contacts of the com­plexes in **1**, and about triple the contribution from H⋯H contacts. Notably, there are no C⋯H/H⋯C contacts for any of the *trans*-Ni^II^(hfac)_2_(H_2_O)_2_ com­plexes in **1**, while the com­plex in **2** has a 4.4% contribution of these contacts to the Hirshfeld surface.

### Comparison to literature structures

**Figure 7:**
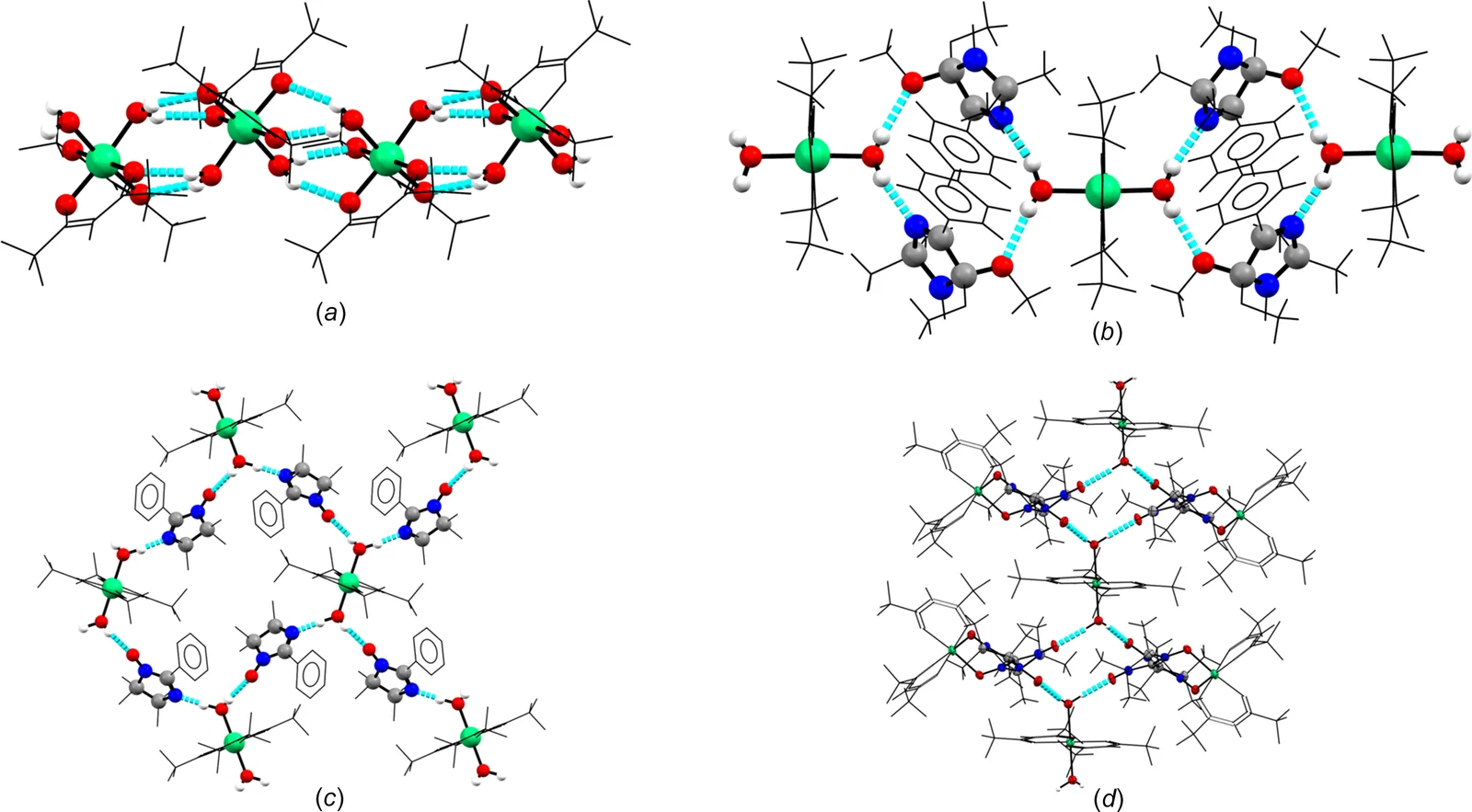
(*a*) Hydrogen bonding between *cis*-Ni^II^(hfac)_2_(H_2_O)_2_ com­plexes in VOXRAB. (*b*) 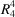
(14) hy­dro­gen-bonded rings in TUKPEU. (*c*) A view of the hy­dro­gen-bond sheet in KUMBEZ. (*d*) 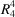
(18) hy­dro­gen-bonded rings in ECOJAI. The CIF files deposited in the CSD for structures VOXRAB, KUMBEZ, and TUKPEU do not con­tain displacement ellipsoid information, so the atoms are drawn as fixed size spheres.

A search of the Cambridge Structural Database (CSD; Version 6.01, including May 2026 updates) (Groom *et al.*, 2016 ▸) reveals only four crystal structures con­taining Ni^II^(hfac)_2_(H_2_O)_2_ com­plexes: VOXRAB (Romero *et al.*, 1992 ▸), KUM­BEZ (Luneau *et al.*, 1992 ▸), TUKPEU (Burdukov *et al.*, 1996 ▸), and ECOJAI (Maekawa *et al.*, 2006 ▸). VOXRAB is a neat form of the *cis*-Ni^II^(hfac)_2_(H_2_O)_2_ com­plex, in which the two aqua ligands are *cis* rather than *trans* across the nickel center. The crystal packing of VOXRAB bears some similarities to that of **1**, with 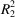
(6) hy­dro­gen-bonded rings between the aqua ligands and hfac ligands leading to chains of metal com­plexes in which the hy­dro­gen-bonded rings are separated by Ni atoms [Fig. 7 ▸(*a*)]. Unlike in crystal **1**, in which the hy­dro­gen-bond network forms two-dimensional sheets separated by F⋯F contacts, the hy­dro­gen-bond chains of metal com­plexes in VOXRAB are one-dimensional, and contacts between chains include F⋯F contacts, as well as F⋯H contacts. An examination of the Hirshfeld surface com­positions between **1** and VOXRAB shows that VOXRAB has significantly more F⋯O contacts com­pared to **1**, while having fewer H⋯O contacts (Table 5 ▸).

The structures of KUMBEZ, TUKPEU, and ECOJAI are all cocrystals of *trans*-Ni^II^(hfac)_2_(H_2_O)_2_ with 2,5-di­hydro­imidazole nitroxide-based coformers. For crystals KUMBEZ and TUKPEU, each H atom on the aqua ligands acts as a donor to a coformer, giving a *trans*-Ni^II^(hfac)_2_(H_2_O)_2_ to coformer ratio of 1:4, similar to what is seen in crystal **2**. Unlike in **2**, the coformers in KUMBEZ and TUKPEU have multiple hy­dro­gen-bond-accepting sites, resulting in the formation of hy­dro­gen-bond networks. In TUKPEU, the hy­dro­gen bonding forms 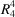
(14) rings in which the aqua ligand acts as a hy­dro­gen-bond donor to an imidazole N atom and a meth­oxy O atom of two separate coformers [Fig. 7 ▸(*b*)]. Similar to **1**, the hy­dro­gen-bond networks in KUMBEZ form two-dimensional hy­dro­gen-bonded sheets parallel to the [101] direction [Fig. 7 ▸(*c*)]. Each *trans*-Ni^II^(hfac)_2_(H_2_O)_2_ com­plex participates in two 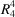
(14) hy­dro­gen-bonded rings, and each hy­dro­gen-bonded ring is separated from the next by the Ni atom of the metal com­plex.

ECOJAI includes a coformer that con­tains a *trans*-Ni^II^(hfac)_2_ center, with a symmetric 2,5-di­hydro­imidazole nitroxide dimer acting as a bidentate ligand on the metal center. This ligand has two hy­dro­gen-bond-accepting sites. Contrary to **2**, in which each H atom of the aqua ligand acts as a donor to both acceptor atoms on the DMB coformer, each acceptor of the coformer in ECOJAI is hy­dro­gen bonded to only one H atom from the aqua ligand. The crystal packing and hy­dro­gen-bond network are quite different from crystal **1**, consisting of 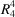
(18) hy­dro­gen-bonded rings in which each H atom of the aqua ligand of the *trans*-Ni^II^(hfac)_2_(H_2_O)_2_ com­plex forms a hy­dro­gen bond with the *N*-oxide O atom of the other com­plex, with a crystallographic inversion center located in the center of the hy­dro­gen-bond network [Fig. 7 ▸(*d*)]. Similarly to TUKPEU, the *trans*-Ni^II^(hfac)_2_(H_2_O)_2_ com­plex participates in two 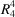
(18) hy­dro­gen-bonded rings, and each hy­dro­gen-bonded ring is separated frem the next by the Ni atom of Ni^II^(hfac)_2_. While the sample size is small, it is observed that cocrystals of *trans*-Ni^II^(hfac)_2_(H_2_O)_2_ are capable of forming different hy­dro­gen-bond motifs depending on the identity of the coformer. Coformers which have two accepting sites on different ends of the mol­ecule can form hy­dro­gen-bonded sheets or rings linked by Ni centers. Water, a coformer with two donating sites and two accepting sites, enabled the formation of sheets which con­tained both ring and chain hy­dro­gen-bond motifs. Notably, the crystal structure of **2** demonstrates that DMB, a coformer with two accepting sites located on the same side of the mol­ecule, prevents the formation of a hy­dro­gen-bond network, even when the DMB mol­ecule itself does not coordinate to the metal center. This provides insight into methods by which cocrystal design can be used to manipulate the hy­dro­gen-bond networks in *trans*-Ni^II^(hfac)_2_(H_2_O)_2_ and structurally related com­pounds.

## Conclusion

In this work, the crystal structures of two solvates of *trans*-Ni^II^(hfac)_2_(H_2_O)_2_ are reported. The crystal structure con­taining water as a coformer formed layers of hy­dro­gen-bond networks separated by layers of CF_3_⋯CF_3_ contacts, a packing motif that potentially results in the macroscopic tendency of the crystals to slip rather than break cleanly when cut. The DMB cocrystal formed discrete units of *trans*-Ni^II^(hfac)_2_(H_2_O)_2_ hy­dro­gen bonded to four DMB mol­ecules, with the DMB mol­ecules occupying all hy­dro­gen-bond-accepting sites of the aqua ligands, preventing the formation of an extended hy­dro­gen-bond network. These new structures increased the amount of reported Ni^II^(hfac)_2_(H_2_O)_2_ crystal structures, with only four such structures having been reported previously. An examination of the hy­dro­gen-bonding motifs in each of the structures revealed that despite the small sample size, *trans*-Ni^II^(hfac)_2_(H_2_O)_2_ cocrystals can form a variety of hy­dro­gen-bond network motifs, and that DMB is capable of disrupting the formation of a hy­dro­gen-bond network in *trans*-Ni^II^(hfac)_2_(H_2_O)_2_ without needing to coordinate to the metal center.

## Supplementary Material

Crystal structure: contains datablock(s) 26169twin5revised, 26164revised, global. DOI: 10.1107/S2053229626009460/vx3026sup1.cif

Structure factors: contains datablock(s) 26169twin5revised. DOI: 10.1107/S2053229626009460/vx30261sup2.hkl

Structure factors: contains datablock(s) 26164revised. DOI: 10.1107/S2053229626009460/vx30262sup3.hkl

Supporting information file. DOI: 10.1107/S2053229626009460/vx30261sup4.mol

Supporting information file. DOI: 10.1107/S2053229626009460/vx30262sup5.mol

CCDC references: 2578544, 2578551

## Figures and Tables

**Table 1:** Experimental details Experiments were carried out at 153 K with Mo *K*α radiation using a Bruker D8 VENTURE diffractometer. H atoms were treated by a mixture of independent and constrained refinement.

	**1**	**2**
Crystal data		
Chemical formula	[Ni(C_5_HF_6_O_2_)_2_(H_2_O)_2_]_3_·4H_2_O	[Ni(C_5_HF_6_O_2_)_2_(H_2_O)_2_]·5C_8_H_10_O_2_
*M*_r_	1598.64	1199.65
Crystal system, space group	Monoclinic, *P*2_1_/*n*	Monoclinic, *P*2_1_/*c*
*a*, *b*, *c* (Å)	24.3968 (14), 7.2381 (4), 31.5461 (17)	11.9278 (5), 15.7842 (6), 15.0115 (6)
β (°)	106.300 (2)	93.887 (1)
*V* (Å^3^)	5346.7 (5)	2819.7 (2)
*Z*	4	2
μ (mm^−1^)	1.25	0.45
Crystal size (mm)	0.27 × 0.25 × 0.08	0.34 × 0.26 × 0.08

Data collection		
Absorption correction	Multi-scan (*TWINABS*; Sevvana *et al.*, 2019 ▸; Sheldrick, 2012 ▸)	Multi-scan (*SADABS*; Krause *et al.*, 2015 ▸)
*T*_min_, *T*_max_	0.603, 0.745	0.679, 0.746
No. of measured, independent and observed [*I* > 2σ(*I*)] reflections	11657, 11657, 10792	66877, 7928, 5947
*R*_int_	Not applicable	0.060
(sin θ/λ)_max_ (Å^−1^)	0.629	0.695

Refinement		
*R*[*F*^2^ > 2σ(*F*^2^)], *wR*(*F*^2^), *S*	0.065, 0.147, 1.16	0.038, 0.106, 1.02
No. of reflections	11657	7928
No. of parameters	1020	558
No. of restraints	566	267
Δρ_max_, Δρ_min_ (e Å^−3^)	0.98, −0.69	0.40, −0.42

Computer programs: *APEX6* (Bruker, 2024 ▸), *SAINT* (Bruker, 2019 ▸), *ShelXle* (Hübschle *et al.*, 2011 ▸), *SHELXT2018* (Sheldrick, 2015*a* ▸), *SHELXL2019* (Sheldrick, 2015*b* ▸), and *Mercury* (Macrae *et al.*, 2020 ▸).

**Table 2:** Hydrogen-bond geometry (Å, °) for **1**[Chem scheme1]

*D*—H⋯*A*	*D*—H	H⋯*A*	*D*⋯*A*	*D*—H⋯*A*
O3_1—H3*OB*_1⋯O1_1^i^	0.81 (2)	2.11 (4)	2.824 (5)	147 (6)
O3_1—H3*OA*_1⋯O4_1^i^	0.81 (2)	2.21 (4)	2.879 (5)	141 (6)
O6_1—H6*OA*_1⋯O1_5	0.80 (2)	2.00 (2)	2.796 (6)	172 (6)
O6_1—H6*OB*_1⋯O4_5^ii^	0.80 (2)	1.94 (2)	2.728 (6)	166 (7)
O3_2—H3*OA*_2⋯O2_5^iii^	0.78*	2.01*	2.786 (6)*	173*
O3_2—H3*OB*_2⋯O3_5^iv^	0.88*	1.87*	2.733 (6)*	167*
O6_2—H6*OB*_2⋯O2_2^ii^	0.81 (2)	2.13 (4)	2.837 (5)	145 (6)
O6_2—H6*OA*_2⋯O5_2^ii^	0.81 (2)	2.18 (4)	2.876 (5)	145 (6)
O3_3—H3*OA*_3⋯O4_5	0.78 (5)	2.07 (5)	2.842 (6)	173 (9)
O3_3—H3*OB*_3⋯O1_5^ii^	0.78 (5)	1.99 (5)	2.766 (6)	173 (8)
O3_4—H3*OB*_4⋯O3_5	0.81 (2)	2.05 (2)	2.842 (6)	168 (8)
O3_4—H3*OA*_4⋯O2_5^iii^	0.81 (2)	1.97 (2)	2.769 (6)	171 (8)
O1_5—H1*OA*_5⋯O2_3^v^	0.81 (2)	2.05 (3)	2.835 (6)	161 (6)
O1_5—H1*OB*_5⋯O1_3^ii^	0.81 (2)	2.31 (5)	2.943 (6)	135 (6)
O2_5—H2*OB*_5⋯O1_4^vi^	0.81 (2)	2.34 (5)	2.965 (6)	135 (6)
O2_5—H2*OA*_5⋯O2_4	0.81 (2)	2.09 (4)	2.845 (6)	154 (6)
O3_5—H3*OA*_5⋯O4_2	0.81 (2)	2.16 (5)	2.823 (6)	139 (6)
O3_5—H3*OB*_5⋯O1_2	0.81 (2)	2.12 (4)	2.848 (6)	150 (6)
O4_5—H4*OA*_5⋯O2_1^ii^	0.82 (2)	2.09 (4)	2.835 (6)	152 (8)
O4_5—H4*OB*_5⋯O5_1^ii^	0.81 (2)	2.10 (5)	2.823 (6)	147 (8)

Symmetry codes: (i) 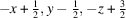; (ii) 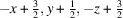; (iii) 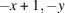, 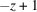; (iv) 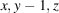; (v) 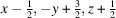; (vi) 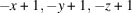. Note: (*) these H atoms were initially placed *via* the difference map. They were refined using fixed positions, as otherwise H3*OB*_2 would refine to an unrealistic position.

**Table 3:** Hydrogen-bond geometry (Å, °) for **2**[Chem scheme1]

*D*—H⋯*A*	*D*—H	H⋯*A*	*D*⋯*A*	*D*—H⋯*A*
O3_1—H1_1⋯O1_2	0.80 (2)	2.17 (2)	2.8978 (17)	152 (2)
O3_1—H1_1⋯O2_2	0.80 (2)	2.33 (2)	2.9854 (17)	140 (2)
O3_1—H2_1⋯O1_3	0.84 (2)	2.31 (2)	2.9100 (16)	128.8 (19)
O3_1—H2_1⋯O2_3	0.84 (2)	2.23 (3)	3.0377 (17)	161 (2)

**Table 4:** Contributions (%) of different contact types to the Hirshfeld surfaces of each of the four *trans*-Ni^II^(hfac)_2_(H_2_O)_2_ com­plexes in **1**, the average values in **1**, and for the *trans*-Ni^II^(hfac)_2_(H_2_O)_2_ com­plex in **2** All contacts include reciprocal contacts. Minor contributions have been omitted, leading to a sum of less than 100% for each com­plex.

Hirshfeld surface contact type	**1**, Residue 1	**1**, Residue 2	**1**, Residue 3	**1**, Residue 4	**1**, Average	**2**
F⋯F	43.8	42.6	45.0	45.1	44.1	0.7
F⋯H/H⋯F	28.9	27.2	28.3	30.2	28.7	61.5
F⋯C/C⋯F	7.3	9.9	6.3	6.0	7.4	2.7
F⋯O/O⋯F	2.2	1.7	2.4	2.6	2.2	1.7
C⋯H/H⋯C	0	0	0	0	0	4.4
H⋯H	3.4	3.5	3.9	3.9	3.7	10.9
O⋯H/H⋯O	12.9	12.4	11.8	12.1	12.3	16.7

**Table 5:** A com­parison of Hirshfeld surface com­positions (%) between **1** and VOXRAB

Hirshfeld surface contact	**1**, average	VOXRAB
F⋯F	44.1	46.6
F⋯H/H⋯F	28.7	25.1
F⋯C/C⋯F	7.4	8.1
F⋯O/O⋯F	2.2	9.8
C⋯H/H⋯C	0	0
H⋯H	3.7	1.8
O⋯H/H⋯O	12.3	8.6
